# Mobile App–Based Self-Report Questionnaires for the Assessment and Monitoring of Bipolar Disorder: Systematic Review

**DOI:** 10.2196/13770

**Published:** 2021-01-08

**Authors:** Eric C Chan, Yuting Sun, Katherine J Aitchison, Sudhakar Sivapalan

**Affiliations:** 1 Department of Psychiatry University of Alberta Edmonton, AB Canada

**Keywords:** mobile apps, mental health, bipolar disorder, smartphone, cell phone

## Abstract

**Background:**

Bipolar disorder is a chronic, progressive illness characterized by recurrent episodes of mania and depression. Self-report scales have historically played a significant role in the monitoring of bipolar symptoms. However, these tools rely on episodic memory, which can be unreliable and do not allow the clinician to monitor brief episodic symptoms or the course of symptoms over shorter periods of time. Mobile app–based questionnaires have been suggested as a tool to improve monitoring of patients with bipolar disorder.

**Objective:**

This paper aims to determine the feasibility and validity of mobile app–based self-report questionnaires.

**Methods:**

We performed a systematic review of the literature according to the PRISMA (Preferred Reporting Items for Systematic Reviews and Meta-Analyses) guidelines. The PubMed, PsycInfo, Web of Science, Ovid MEDLINE, and EMBASE databases were searched for papers published in English that assessed adherence to and the validity of mobile app–based self-report questionnaires. Relevant studies published from database creation to May 22, 2020, were identified, and results examining the validity of and rates of adherence to app-based self-report questionnaires are reported.

**Results:**

A total of 13 records were identified for inclusion in this review. Of these studies, 4 assessed the concurrent validity of mobile app–based self-report tools, with the majority of findings indicating significant associations between data collected using these tools and the Young Mania Rating Scale, Hamilton Depression Rating Scale-17, or Montgomery-Åsberg Depression Rating Scale (*P*<.001 to *P*=.24). Three studies comparing the variability or range of symptoms between patients with bipolar disorder and healthy controls suggested that these data are capable of differentiating between known groups. Two studies demonstrated statistically significant associations between data collected via mobile app–based self-report tools and instruments assessing other clinically important factors. Adherence rates varied across the studies examined. However, good adherence rates (>70%) were observed in all but 1 study using a once-daily assessment. There was a wide range of adherence rates observed in studies using twice-daily assessments (42%-95%).

**Conclusions:**

These findings suggest that mobile app–based self-report tools are valid in the assessment of symptoms of mania and depression in euthymic patients with bipolar disorder. Data collected using these tools appear to differ between patients with bipolar disorder and healthy controls and are significantly associated with other clinically important measures. It is unclear at this time whether these tools can be used to detect acute episodes of mania or depression in patients with bipolar disorder. Adherence data indicate that patients with bipolar disorder show good adherence to self-report assessments administered daily for the duration of the study periods evaluated.

## Introduction

Bipolar disorder is a chronic, progressive illness characterized by recurrent episodes of mania and depression. The international 12-month prevalence of bipolar I disorder is 0.0% to 0.6%, and the international 12-month prevalence of bipolar II disorder is 0.3% [1]. Both manic and depressive episodes are associated with impairments in social and occupational functioning, and the World Health Organization’s World Mental Health Surveys identified the disorder as having the second-strongest effect on days out of role compared with other common physical and mental illnesses [2-5]. In addition, bipolar disorder is associated with a high risk of suicide, with one-third to one-half of patients attempting suicide at least once in their lifetime and 15% to 20% of suicide attempts completed [6]. Given such adverse consequences of mania and depression, timely detection of relapse is an important aspect in the psychiatric care of the disease.

No biomarker has been approved for the diagnosis or assessment of bipolar disorder, so medical practitioners must rely on clinical assessment and reports from the patient and collateral sources in order to monitor the disease. However, detection of mood episodes can be delayed, with previous data indicating that the interval between illness onset and hospitalization is often 3 weeks or more [7]. One challenge for the detection of mood episodes is the lack of insight that can occur in patients with bipolar disorder, especially during episodes of pure mania [8]. Previous data suggest, however, that some patients in acute mania may retain awareness of their diagnosis and its potential consequences despite having impaired insight into their current symptoms [9]. Given patients’ preserved awareness of their diagnosis even in the context of active symptoms, the use of self-report questionnaires has the potential to facilitate symptom monitoring, including changes over time.

Self-report scales, such as the Mood Disorder Questionnaire (MDQ) and the Altman Self-Rating Mania Scale (ASRM), have previously been developed for use in the monitoring of bipolar symptoms. These scales have been validated in inpatient populations with bipolar disorder, with respective sensitivities and specificities of 86% and 71% for the MDQ and 93% and 33% for the ASRM [10-12].

Traditionally, self-report scales have been administered via pen and paper; however, some limitations exist with this form of data collection. When administered in the context of visits with a health care provider, these tools rely on retrospective reporting of symptoms, which can be unreliable and do not allow the clinician to monitor symptoms associated with brief mood episodes or the course of symptoms over shorter periods of time [12-14]. In a study asking participants to complete paper diaries on a daily basis, participants were found to record entries outside of the requested time frame and inaccurately report the date of these entries, reducing the accuracy of the data collected [15]. In addition, the frequency with which the clinician is able to review responses obtained via pen and paper is limited by the frequency in which the responses are forwarded to the provider. This often occurs on clinic visits, which limits the ability of the health care provider to respond in a timely fashion if the patient deteriorates between scheduled appointments.

The administration of self-report scales using mobile apps has the potential to circumvent some of these issues. Automatic transmission of data using a mobile device could allow clinicians to monitor symptoms in real time, improving their ability to proactively detect and engage the patient when symptoms relapse. In addition, scale administration using a mobile app may be less disruptive for the patient, increasing the frequency that the patient is willing to complete the scale. For example, one study described a mobile app for monitoring nonaffective psychosis that yielded more data points and took less time compared with the text messaging–only equivalent [16]. The increased data collection afforded by the use of mobile apps may also have uses in research settings. Frequent administration of scales may allow researchers to better characterize the course of illness over time and to identify warning signs that mark early deterioration.

Given the variability in the course of symptoms in bipolar disorder, the use of mobile apps in this population has been of considerable recent interest, with 35 apps identified using the Google Play and iOS stores in a previous systematic review [17]. Studies have shown that 60% to 70% of patients with mental illness would be interested in using a mobile app to monitor their mental health condition, and a study examining publicly available consumer reviews of 48 apps for bipolar disorder, the majority of which were symptom-monitoring apps (1911/2173, 87.9%), found that 1608 of 2173 (74.0%) reviews included positive appraisals of the app discussed [13,18-20]. Additionally, a recent study evaluating 2 smartphone-based self-monitoring systems for bipolar disorder showed acceptable usefulness, usability, feasibility, and technical stability for both systems evaluated [21]. However, a 2015 review showed that 60% of symptom-monitoring apps available did not use validated screening measures [17]. Furthermore, it is possible that for a given validated screening tool, data collected via a mobile app may differ from those collected via a pen-and-paper version.

The validity of a scale is defined as “the extent to which an instrument indeed measures the latent dimension or construct it was developed to evaluate” [22]. The major forms of validity are content validity, criterion validity, and construct validity. Content validity refers to whether the measure adequately assesses the domain of interest, and it is primarily assessed through evaluation by experts and the target population. Criterion validity refers to whether the results of a measure relate to another measure of relevance. It includes predictive validity (the ability of the measure to predict a future result or answer a future question) and concurrent validity (the strength of the relationship between the new measure and a gold standard measurement made at a similar time). Construct validity refers to the degree to which the measure assesses the construct of concern. Construct validity can be evaluated through convergent validity, discriminant or divergent validity, differentiation or comparison between known groups, or correlational analysis [22].

The aim of this systematic review was to assess the feasibility and validity of self-report questionnaire-based mobile apps as tools for bipolar symptom monitoring through a systematic review of the literature. We identified studies in which patients with bipolar disorder were monitored using self-report scales administered by a mobile app with or without comparison to a traditional form of symptom monitoring, such as pen-and-paper rating scales or standardized clinician interviews. The outcomes of interest in this review were adherence rates and the criterion or construct validity of self-report scales administered by mobile app.

## Methods

In order to identify data describing the feasibility and validity of mobile apps in the assessment of bipolar disorder, we conducted searches of the PubMed, PsycInfo, Web of Science, Ovid MEDLINE, and EMBASE databases. One researcher (YS) searched these databases using the following keywords: “mental disorders,” “psychiatry,” or “mental health” AND “mobile application,” “cell phone,” or “smartphone,” excluding the term “substance-related disorders.” All records published in English listed from database creation to May 22, 2020, were identified. In addition, the references on the full paper of the records assessed were reviewed in order to identify other potential candidates for inclusion.

YS and ECC independently screened the records to identify papers suitable for inclusion in this review. In the case of disagreement between the 2 authors, records were evaluated by a third author (SS), who determined whether the paper would be forwarded to the next step of screening. There was no disagreement between authors following the review of the full papers.

Titles and abstracts of records were screened using the following exclusion criteria: (1) the study did not refer to the use of mobile apps, smartphones, or mobile phone or technology as the primary intervention of interest, or the intervention of interest was solely text message based; (2) bipolar disorder was not the primary condition of interest; (3) the interventions studied did not include self-report symptom monitoring as a component; and (4) the study did not present data from an applied intervention (such as a protocol paper, review paper, or response or correction to another paper).

The full text of the remaining studies were evaluated, and studies were excluded if they met one of the following criteria: (1) the study did not present data on adherence or validity; (2) the study did not present data from an applied intervention (such as a protocol or review paper); (3) the study did not refer to symptom assessment via self-report by mobile app, smartphone, or mobile phone or technology as a primary intervention of interest; (4) the intervention of interest was solely text message based; and (5) bipolar disorder was not the primary condition of interest.

Studies identified for inclusion in this review were then evaluated for data on the adherence rates and validity of mobile app–based symptom monitoring tools with or without comparison to standardized pen-and-paper or clinical interview–based measures. ECC and YS assessed each of the identified studies for bias using the Cochrane Risk of Bias 2 tool or the Cochrane Risk of Bias in Non-Randomized Studies of Interventions assessment tool. These tools were developed for the assessment of bias in randomized and nonrandomized studies, respectively [23,24]. These assessments were reviewed by another author (SS) and are available in Multimedia Appendix 1.

## Results

### Identified Records

**Figure 1:**
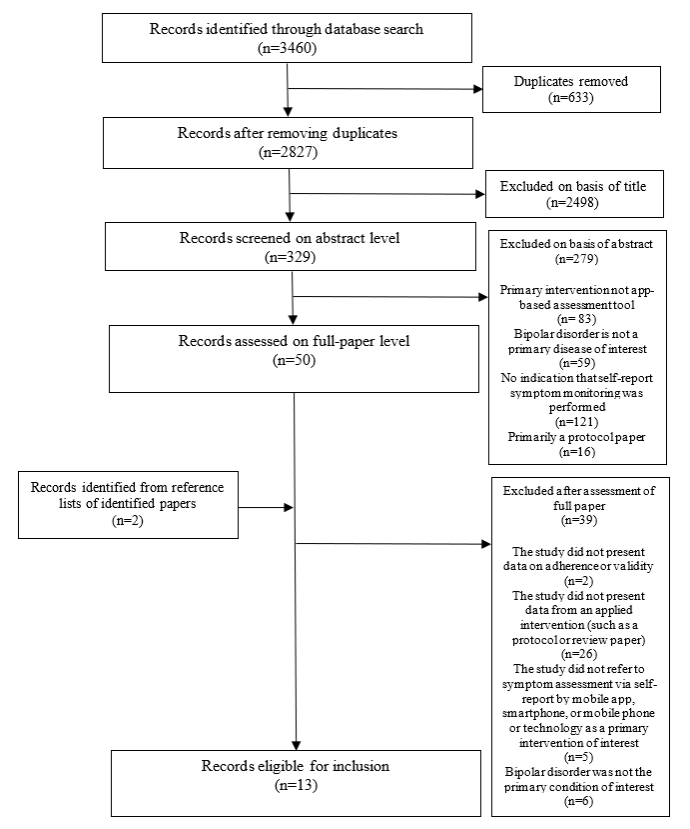
PRISMA (Preferred Reporting Items for Systematic Reviews and Meta-Analyses) flow diagram.

The flow diagram of the search method is depicted in Figure 1. Initial searches produced 2827 unique records following the removal of duplicates. A total of 50 records were identified following screening of the abstracts, and their references were also searched for further relevant studies. Following the search procedure described above, 13 records were identified for inclusion in this review; study characteristics are listed in Table 1. Findings of each study are listed separately (Table 2). The assessments of the risk of bias are described in Multimedia Appendix 1.

**Table 1:** Characteristics of included studies.

Reference	Location	Participants, n	Mobile app–based intervention	Comparison (if applicable)	Duration
Busk et al (2020) [25]	Copenhagen, Denmark	84	Monsenso system: self-monitoring of 10 symptoms completed daily.	YMRS^a^ and HDRS^b^ at baseline and after 4 weeks, 3 months, 6 months, and 9 months.	9 months
Carr et al (2018) [26]	Oxford, United Kingdom	43 (bipolar disorder);	Mood Zoom smartphone app: 6-item assessment of mood and related items administered 10 times daily.	N/A^c^	3 months
		26 (borderline personality disorder);			
		44 (healthy controls)			
Depp et al (2012) [27]	San Diego, CA	18 (intervention)	9-point bipolar anchored scale completed twice per day. Could not be completed after 2 hours.	Daily paper-and-pencil mood charts.	12 weeks
		22 (comparison)		MADRS^d^ and YMRS completed at baseline and 6 weeks and 12 weeks after baseline.	
Depp et al (2015) [28]	San Diego, CA	51 (intervention), (41 analyzed);	PRISM: 10 questions followed by rating of current mood state on a 9-point bipolar anchored scale completed twice per day.	Daily pencil-and-paper mood charts.	10 weeks
		53 (comparison), (41 analyzed)			
Faurholt-Jepsen et al (2015) [29]	Copenhangen, Denmark	30	MONARCA: self-monitoring of 11 symptoms completed daily. Allowed for retrospective data entry up to 2 days later.	Monthly clinical assessment via HDRS-17 and YMRS.	6 months
				Scores compared to those obtained via app from day of assessment and 3 previous days.	
Faurholt-Jepsen et al (2015) [30]	Copenhagen, Denmark	39 (intervention);	MONARCA: self-monitoring of 11 symptoms completed daily. Allowed for retrospective data entry up to 2 days later.	Participants provided with a smartphone without the MONARCA system.	6 months
		39 (comparison)			
Faurholt-Jepsen et al (2019) [31]	Copenhagen, Denmark	84 patients (participants in MONARCA II trial)	Monsenso app for daily self-monitoring of mood, mixed mood, and irritability level.	HDRS, YMRS, FAST^e^, PSS^f^, and WHOQoL^g^ carried out at 4 weeks, 3 months, 6 months, and 9 months.	9 months
Faurholt-Jepsen et al (2019) [32]	Copenhagen, Denmark	84 patients with bipolar disorder (participants in MONARCA II trial)	Monsenso app for daily self-monitoring of mood and related symptoms.	No comparison used for outcomes of interest.	9 months
Hidalgo-Mazzei et al (2016) [33]	Barcelona, Spain	51	SIMPLe app: short 5-item screening tests completed daily.	N/A	3 months
			Weekly yes/no questions for DSM-5^h^ criteria of manic and depressive episodes.		
Hidalgo-Mazzei et al (2018) [34]	Barcelona, Spain	201	SIMPLe 1.5 (improved version of SIMPLe 1.0): short 5-item screening tests completed daily.	N/A	6 months
			Weekly Yes/No questions for DSM-5 criteria of manic and depressive episodes.		
			Additional features included medication reminders, personalized prodromal symptoms, gamification module, mood chart sharing, and psychoeducational messages.		
Li et al (2019) [35]	Hershey, PA	10 (bipolar disorder);	Twice-daily mood and stress self-report, once daily sleep measures.	N/A	14 days
		10 (healthy controls)			
Saunders et al (2017) [36]	Oxford, United Kingdom	21	Mood Zoom app: daily mood monitoring.	N/A	12 weeks
			True Colours system: weekly mood measures.		
Schwartz et al (2016) [37]	Pennsylvania	10 (bipolar I or II);	4 items on visual analog scale and 1 item on Likert scale completed twice per day.	N/A	2 weeks
		10 (healthy controls)			

^a^YMRS: Young Mania Rating Scale.

^b^HDRS: Hamilton Depression Rating Scale.

^c^N/A: not applicable.

^d^MADRS: Montgomery-Åsberg Depression Rating Scale.

^e^FAST: Functional Assessment Short Test.

^f^PSS: Perceived Stress Scale.

^g^WHOQoL: World Health Organization Quality of Life (abbreviated).

^h^DSM-5: Diagnostic and Statistical Manual of Mental Disorders, fifth edition.

**Table 2:** Summary of findings on mobile app use in bipolar symptom monitoring.

Reference	Completion rates	Correlation between data obtained via mobile app and comparator	
Busk et al (2020) [25]	Average self-assessment adherence: 82.8%	Mood scores:	
			HDRS^a^: *r*=–0.40; *P*<.001; YMRS^b^: *r*=0.22; *P*<.001
Carr et al (2018) [26]	20/43 (47%) of patients with bipolar disorder; 14/26 (54%) of patients with borderline personality disorder; 20/44 (45%) of healthy controls had satisfactory data; 14/26 (54%) of patients with borderline personality disorder	Variability of negative mood:	
			BD^c^ median: –0.99 (IQR 0.85); BPD^d^ median: 1.71 (IQR 1.11); Healthy control median: 0.35 (IQR 0.47) BD vs BPD (FDR^e^): 1.57 × 10^–3^; BD vs HC (FDR): 2.31 × 10^–2^
		Variability of positive mood:	
			BD median: –0.91 (IQR 0.70); BPD median: 1.42 (IQR 0.56); Healthy control median: 0.62 (IQR 0.52) BP vs BPD (FDR): 1.21 × 10^–3^; BP vs HC (FDR): 6.13 × 10^–1^ (nonsignificant)
		Variability of irritability:	
			BD median: –0.56 (IQR 0.43); BPD median: 1.01 (IQR 0.49); Healthy control median: 0.33 (IQR 0.46) BP vs BPD (FDR): 1.87 × 10^–3^; BP vs HC (FDR): 2.39 × 10^–2^
Depp et al (2012) [27]	Intervention: 42.1%; Comparison: 82.9%; *t*_35_=5.8; *P*<.001	**Mood ratings:**	
			Intervention:
			MADRS^f^: *r*=–0.567; *P*=.01 YMRS: *r*=0.294; *P*=.24
			Comparison:
			*r*=–0.243; *P*=.35 *r*=0.452; *P*=.07
Depp et al (2015) [28]	Intervention: 65%; Comparison: 83%	—^g^	
Faurholt-Jepsen et al (2015) [29]	—	Mood:	
			HDRS-17: β=–0.058; *P*<.001 YMRS: β=0.039; *P*<.001
		Sleep:	
			HDRS-17: β=0.02; *P*=.21 YMRS: β=–0.047; *P*=.03
		Activity:	
			HDRS-17: β=–0.042; *P*<.001 YMRS: β=0.048; *P*<.001
		Stress:	
			HDRS-17: β=0.046; *P*<.001 YMRS: β=0.012; *P*=.35
Faurholt-Jepsen et al (2015) [30]	Intervention: 93.03% (7.15% done retrospectively)	—	
Faurholt-Jepsen et al (2019) [31]	Adherence rate: 72.6%	Mood ratings:	
			HDRS: β=–0.033; *P*<.001 YMRS: β=0.044; *P*<.001
		Self-reported mixed symptoms:	
			Clinically rated mixed symptoms: β=3.40; *P*=.02 PSS^h^: β=14.08; *P*<.001 WHOQoL^i^: β=–7.80; *P*=.15 FAST^j^: β=–2.02; *P*=.72
		Irritability:	
			YMRS: β=0.023; *P*<.001 PSS: β=11.32; *P*<.001 WHOQoL: β=–11.59; *P*<.001 FAST: β=–9.90; *P*<.001
Faurholt-Jepsen et al (2019) [32]	Reported in previous study [29]	Mood instability factor (number of mood changes over period evaluated by scale):	
			FAST: β=–12.04; *P*<.001 PSS: β=10.52; *P*<.001 WHOQoL: β=–12.17; *P*<.001
Hidalgo-Mazzei et al (2016) [33]	88% completion rate; 74% of users actively using app after 3 months	—	
Hidalgo-Mazzei et al (2018) [34]	70/201 (35%) users dropped out during the first month; 30% of participants using the app regularly after 6 months	—	
Li et al (2019) [35]	70% completion rate in bipolar patients and healthy controls	**Variability of symptoms:**	
			Mood:
			Bipolar ICC^k^: 0.55; healthy control ICC: 0.72; *P*<.001
			Energy:
			Bipolar ICC: 0.49; healthy control ICC: 0.61; *P*<.001
			Speed of thoughts:
			Bipolar ICC: 0.40; healthy control ICC: 0.67; *P*<.001
			Impulsivity:
			Bipolar ICC: 0.16; healthy control ICC: 0.68; *P*<.001
			Sleep:
			Bipolar ICC: 0.46; healthy control ICC: 0.30; *P*<.001
Saunders et al (2017) [36]	Daily questionnaire: median 86.67%; Weekly questionnaire: median 100%	—	
Schwartz et al (2016) [37]	Bipolar: 95%; Controls: 88%; *P*=.68	14-day mean of mood:	
			Bipolar median: 48.6; control median: 53.2; *P*=.04
		14-day mean of energy:	
			Bipolar median: 44.7; control median: 52.1; *P*=.007
		14-day range of mood:	
			Bipolar median: 48.0; control median: 32.5; *P*=.04
		14-day range of thoughts:	
			Bipolar median: 59.5; control median: 26.5; *P*=.002
		14-day range of impulsivity:	
			Bipolar median: 76; control median: 28.5; *P*=.005

^a^HDRS: Hamilton Depression Rating Scale.

^b^YMRS: Young Mania Rating Scale.

^c^BD: bipolar disorder.

^d^BPD: borderline personality disorder.

^e^FDR: false discovery rate.

^f^MADRS: Montgomery-Åsberg Depression Rating Scale.

^g^Not available.

^h^PSS: Perceived Stress Scale.

^i^WHOQoL: World Health Organization Quality of Life (abbreviated).

^j^FAST: Functional Assessment Short Test.

^k^ICC: intraclass correlation coefficient.

### Data on Validity

A total of 4 papers identified for inclusion assessed the concurrent validity of mobile app–based self-report tools, all compared against the Young Mania Rating Scale (YMRS) and either the Hamilton Depression Rating Scale (HDRS) or the Montgomery-Åsberg Depression Rating Scale (MADRS) [25,27,29,31]. All 4 studies found a statistically significant association between mood ratings collected via self-report using a mobile app and clinical assessment using the HDRS or MADRS. In addition, 3 studies found a statistically significant association between mood ratings collected via self-report using a mobile app and clinical assessment using the YMRS [25,29,31]. The fourth study, however, did not observe a statistically significant relationship [27]. One study also found a statistically significant relationship between self-reported mixed symptoms and clinically rated mixed symptoms, as well as a statistically significant relationship between self-reported irritability and YMRS scores [31]. One study examined mood ratings that were reported using a paper-and-pencil tool as well [27]. They did not find a statistically significant correlation between mood ratings reported using a paper-and-pencil tool and either the MADRS or YMRS [27].

A total of 3 studies examined the ability of self-report scales administered via a mobile app to differentiate between known groups, a form of construct validity [26,35,37]. Of these, 2 studies evaluated the differences in the variability of symptoms (mood, irritability, energy, speed of thoughts, impulsivity, or sleep) between patients with bipolar disorder and healthy controls [26,35]. These studies found statistically significant differences in the variability of symptoms between the 2 groups, with the exception of variability of positive mood [26]. One study also compared the variability of negative mood, positive mood, and irritability between patients with bipolar disorder and patients with borderline personality disorder; this study observed a statistically significant difference between the 2 groups for all 3 variables studied [26]. One study examined the difference in the 14-day mean of participants’ mood and energy, as well as the 14-day range of mood, thoughts, and impulsivity between patients with bipolar disorder and healthy controls [37]. Statistically significant differences were observed between the 2 groups for all 5 of these variables [37].

Additionally, 2 studies examined the convergent validity of self-report symptom assessments administered via a mobile app with instruments assessing related factors: the Functional Assessment Short Test (FAST), the Cohen Perceived Stress Scale (PSS), and the abbreviated World Health Organization Quality of Life scale (WHOQoL-BREF) [31,32]. A statistically significant relationship was observed between self-reported mixed symptoms and PSS scores, but not with WHOQoL-BREF or FAST scores [31]. A statistically significant association was observed for both irritability and mood instability determined using self-report compared with the FAST, PSS, and WHOQoL-BREF [31,32].

### Data on Adherence

Varying levels of adherence to the reporting protocol, ranging from 42% to 95%, were reported among studies in which measures were administered once or twice daily, with all but 1 study that used once-daily administration having adherence rates >70% [25,27,28,30,31,33,35-37]. Two studies reported high dropout rates [26,34]. In 1 study, participants were asked to complete a 6-item assessment 10 times daily, with 59 out of 113 (52.2%) of participants dropping out across all 3 study groups [26]. The other study reported that 70 out of 201 (34.8%) participants dropped out during the first month, which was higher than the percentage of participants dropping out in another study using a similar mobile app [33,34]. Compliance rates were substantially higher for the paper-and-pencil conditions in the 2 studies reported by Depp et al [27,28]. However, the frequency of measure completion was not the same between the 2 groups, and the paper-and-pencil condition could complete the measure at any time, whereas the phone condition was time limited [27,28]. These differences may have contributed substantially to the differences in completion rates between conditions.

## Discussion

### Principal Findings

The overall results of this review suggest that mobile app–based self-report questionnaires demonstrate concurrent validity when compared with established measures of depression and mania and convergent validity when compared with other related assessment tools. Furthermore, current evidence indicates that mobile app–based self-report questionnaires are able to differentiate between patients with bipolar disorder and patients with borderline personality disorder or healthy controls. In terms of protocol adherence, variability was observed in completion rates, with higher overall adherence rates in participants completing questionnaires daily compared with twice daily. High dropout rates were observed when participants were asked to complete the measure 10 times per day.

In this review, 4 studies analyzed the association between the self-reporting of symptoms via a mobile app and clinical assessment tools. While all 4 studies found a statistically significant association between mood ratings collected via self-report and clinical assessment tools for depression, only 3 out of 4 studies found a statistically significant association between mood ratings collected via self-report and the YMRS. Of note, the study in which no statistically significant correlation was found compared YMRS scores to data collected over the entire study duration and to those collected during the first 6 weeks of the study [27]. As the YMRS assesses symptoms over the preceding 48 hours, the poor correlation may be at least partly attributable to the difference in time periods observed. Only 1 other study reported the period of data used in the comparison, comparing YMRS scores to data collected over the preceding 3 days [29]. This may be a more appropriate comparison, especially as one goal of app-based self-report scales is the detection of acute mood states and changes in symptoms over time.

Furthermore, data collected via the paper-and-pencil condition did not have a statistically significant correlation with either the MADRS or YMRS [27]. This suggests that app-based self-report scales may more accurately collect data on depressive symptoms compared with their paper-based counterparts. While there are few data comparing mobile assessments with rating scales administered via paper and pencil, it has been suggested elsewhere that participants may be more forthcoming when reporting symptoms through mobile assessments [38]. In addition, it has been shown that participants completing measures via paper and pencil may complete the entries retrospectively and hence, outside the specified time frame being assessed [17]. This may explain the seemingly increased accuracy of symptoms reported via app-based measures compared with paper and pencil.

A manic or depressive episode at study onset was an exclusion criterion for many of the studies identified [27-33]. In addition, 3 other studies indicated that patients were euthymic for the duration of the study [25,26,36]. The remaining studies did not state whether any participants experienced acute episodes of mania or depression. As such, it is unclear whether mobile app–based self-report tools can detect acute mood episodes in patients with bipolar disorder.

Some studies assessed the ability of mobile app–based self-report tools to differentiate between known groups [26,35,37]. These studies found statistically significant differences between patients diagnosed with bipolar disorder and healthy controls. While differences in mean mood and mean energy were observed between the 2 groups in 1 study, the magnitude of the difference in range of thoughts and range of impulsivity between the 2 groups was higher [37]. The 2 other studies comparing 2 known groups also observed differences in the variability of symptoms associated with bipolar disorder [26,35]. These findings suggest that the range and course of symptoms measured using mobile app–based self-report tools may allow us to distinguish patients with bipolar disorder from healthy controls.

Studies comparing data collected via self-report assessments administered via a mobile app to the FAST, PSS, and WHOQoL-BREF observed statistically significant associations between some data collected and these measures. As the FAST, PSS, and WHOQoL-BREF assess functional impairment, psychological distress, and quality of life, these findings suggest that data collected via self-report using a mobile app may also reflect other factors of clinical importance [39-41].

Lower rates of adherence to the protocol were observed in most studies in which assessments were administered twice daily compared with studies in which assessments were administered once daily. Furthermore, 1 study in which assessments were administered 10 times per day observed high dropout rates during its 3-month course [26]. These findings suggest that users may have difficulty completing multiple assessments per day but are able to manage assessments occurring once daily. Different proportions of participants dropped out in 2 studies administering similar mobile apps [33,34]. The reason for this is unclear. Previous data indicate that users value apps that are simple and intuitive to use [42]. The study in which higher dropout rates were observed used a version of the app containing numerous additional features, so it is possible that users found the app more complicated and were less willing to continue regular use as a result [34].

### Limitations

In this review, only English studies from peer-reviewed journals were considered. As very few (n=49) non-English papers were identified prior to screening, this was felt to have minimal impact on overall results. As there were large numbers of protocol papers identified, for which it is not possible to exclude unpublished data, it is also possible that publication bias may have resulted in missed negative findings. While 13 papers were identified for inclusion in this review, only 5 different research groups seem to be represented, based on the names and affiliations of authors. One group is represented in 5 studies, which is over one-third of those identified for inclusion [25,29-32]. This may contribute to bias; however, it is reassuring that the reported findings appear to be fairly consistent across the different groups included. As noted above, no study reported on the ability of mobile app–based self-report tools to detect acute mood episodes. As such, it is unclear whether these tools are suitable for this purpose.

### Future Research

Further studies on the validity of mobile app–based assessment tools, especially studies evaluating the ability of these tools to detect acute mood states, will better inform us about the potential utility of these tools in clinical settings. Future research into the course of symptoms measured using these tools may also provide insights into the differences between patients with bipolar disorder and healthy controls. Furthermore, the use of repeated self-report questionnaires combined with physiological and behavioral monitoring, which have been examined elsewhere [43], and with other biomarkers also bears further investigation and may further our understanding of bipolar disorder.

### Conclusions

These findings suggest that mobile app–based self-report tools are valid in the assessment of symptoms of mania and depression in euthymic patients with bipolar disorder. These findings also suggest that data on the range and variability of symptoms collected using a mobile app differ between patients with bipolar disorder and healthy controls and are significantly associated with other clinically important measures. It is unclear at this time whether these tools can be used to detect acute episodes of mania or depression in patients with bipolar disorder. Adherence data indicate that patients with bipolar disorder show good adherence to self-report assessments administered daily for the duration of the study periods evaluated.

## Appendix

Multimedia Appendix 1 Assessment of risk of bias in included studies.

## Abbreviations

ASRM: Altman Self-Rating Mania Scale
FAST: Functional Assessment Short Test
HDRS: Hamilton Depression Rating Scale
MADRS: Montgomery-Åsberg Depression Rating Scale
MDQ: Mood Disorder Questionnaire
PSS: Cohen Perceived Stress Scale
WHOQoL-BREF: abbreviated World Health Organization Quality of Life
YMRS: Young Mania Rating Scale
