# Supplementary material for: Mobile App–Based Self-Report Questionnaires for the Assessment and Monitoring of Bipolar Disorder: Systematic Review
Source: JMIR Form Res. 2021 Jan 8;5(1):e13770. doi: 10.2196/13770 (PMC7822726; doi:10.2196/13770)
Supplement: Multimedia Appendix 1 [file formative_v5i1e13770_app1.pdf]

Appendix 1. Assessment of risk of bias in included studies

Reference; Location	Risk of Bias
Busk et al. (2020) [25]<br><br>Copenhagen, Denmark	Patients had low prevalence of severe symptoms which prevents the data from assessing how the method performs in assessing severe cases.<br><br>This was felt to have had moderate impacts on the results.
Carr et al. (2018) [26]<br><br>Oxford, UK	Must be comfortable with navigating device, otherwise excluded. All group participants must be 18 years of age or older.<br><br>Bipolar participants and Borderline personality participants were taking a range of different medications with varying degrees of number of medications per participant. Healthy control participants were not taking any medication.<br><br>Recordings shorter than 4 days were discarded and 22 reports were discarded because devices failed to record data.<br><br>These factors had the potential to cause significant impact on the results.
Depp et al. (2012) [27]<br><br>San Diego, USA	Patients with recent substance use disorder, hospitalization, MADRS >32 or YMRS >20 were excluded.<br><br>Completion rates: The frequency of scale completion differed between groups. The mobile phone condition was time-limited but the paper-and-pencil condition was not, which could also lead to retrospective data entry. The authors report on both compliance rates including and excluding the missing data from 3 participants in the comparison group.<br><br>Reported symptoms: Mean mood ratings over study duration and over first 6 weeks were used in comparison to MADRS and YMRS, however MADRS and YMRS are conducted at discrete points in time, weakening potential correlation of individual measurements in both mobile phone and paper-and-pencil conditions.<br><br>These factors were felt to have potential substantial impact on the results.
Depp et al. (2015) [28]<br><br>San Diego, USA	Patients with recent substance use disorder, hospitalization, MADRS >32 or YMRS >20 were excluded.<br><br>The frequency of scale completion differed between groups. The mobile phone condition was time-limited but the paper-and-pencil condition was not, which could also lead to retrospective data entry.<br><br>These factors may have a significant impact on relative completion rates.
Faurholt-Jepsen et al. (2015a) [29]<br><br>Copenhangen, Denmark	Participants lacking the technical knowledge to use the smartphone and with HDRS or YMRS score >17 were excluded.<br><br>In addition, users completing the measure shortly prior to clinical interview with HDRS/YMRS may provide similar responses even if their symptoms have changed in the interim to show consistency.<br><br>Overall, these limitations were felt to potentially have moderate impact on the results.

Faurholt-Jepsen et al. (2015b) [30]<br><br>Copenhagen, Denmark	Participants lacking the technical knowledge to use the smartphone and with HDRS or YMRS score >17 were excluded.<br><br>These factors may have a moderate impact on completion rates, however if completion rates were to decrease in depressed and manic episodes, this may also assist practitioners in intervening.
Faurholt-Jepsen et al. (2019a) [31]<br><br>Copenhagen, Denmark	Patients with schizophrenia, schizotypal or delusional disorders, previous use of the MONARCA system, pregnancy and lack of Danish language skills were excluded. Patients must be minimum 18 years of age and have been previously treated at a specialized mood disorder clinic for 2 years.<br><br>During treatment, participants have been treated with mood stabilizers and thus, had a few medication changes during study period. Patients included were in full or partial remission.<br><br>Reporting of symptoms on the comparison measures may have been influenced by the participants' knowledge of their recent responses on the mobile application, potentially increasing the association between the two forms of data collection.<br><br>The impact of the above factors could pose a significant effect on results.
Faurholt-Jepsen et al. (2019b) [32]<br><br>Copenhagen, Denmark	Patients with schizophrenia, schizotypal or delusional disorders, previous use of the MONARCA system, pregnancy and lack of Danish language skills were excluded. Include patients with an HDRS score ≤ 17 or a YMRS score ≤ 17. Patients must also be comfortable with mobile technology use.<br><br>Patients included in study had previously received treatment for 2 years in a specialized mood disorder clinic. However, the period of 9 months may not capture the full picture of long-term increased stress sensitivity. In addition, data related to external factors such as familial support or financial resources were not included.<br><br>These factors could pose moderate impacts on completion rates but more significant impacts on resulting data.
Hidalgo-Mazzei et al. (2016) [33]<br><br>Barcelona, Spain	Participants with IQ <90, HDRS ≥8, YMRS ≥6, or without the requisite technical skills were excluded.<br><br>A specific brand of smartphone was required for study inclusion, potentially causing socioeconomic status to be a confounder. The authors report on demographic data suggesting this may not be the case, but do not stratify participants by income.<br><br>Users also received a brief psychoeducational message after measure completion, this positive feedback may encourage increased compliance.<br><br>These factors were felt to have a moderate impact on rates of scale completion.
Hidalgo-Mazzei et al. (2018) [34]<br><br>Barcelona, Spain	Patients who are under 18 years of age, not receiving direct and routine pharmacological treatment, do not speak fluent Spanish and who do not own or use a compatible smartphone were excluded.<br><br>Requires a specific branding of phone. Daily pop-up notification prompts participant to read a psychoeducational message providing brief information to cope with situations to avoid relapses.<br><br>Retention factors were likely related to its entire online delivery where screening, enrolment and follow-up were done online without any face-to-face or telephone

	contact. The intent was to give participants a sense of self-management and privacy, however, this could also contribute to less commitment to the programme. The accuracy and the reliability of screening, baseline and follow-up data could not be verified.<br><br>Many participants were also receiving psychological treatment which may influence retention and data collection. For example, the additional treatment may have increased adherence rates or dropout from the psychological treatment program may have led to cessation of application use. Gamification was also cited to be influential in the retention and data collection, however, the other new additions may have adversely affected adherence rates.<br><br>These factors were felt to potentially have a significant impact on rates of completion as well as the resulting data collection.
Li et al. (2019) [35]<br><br>Hershey, USA	All participants in the Bipolar Disorder Group must be a minimum age of 18 and diagnosed with BD type I or II by the MINI 5.0.<br><br>All participants in the Healthy Control Group must be a minimum age of 18, have no family history of any major psychiatric illness and no personal history of major mood, anxiety or psychotic psychiatric illness as determined by MINI 5.0<br><br>Participants must be comfortable using the smartphone provided to them and must be fluent in English. Both groups of participants did not differ significantly on age, gender and employment status, small sample lacked diversity.<br><br>These factors could have moderate impact on rates of completion of intervention
Saunders et al. (2017) [36]<br><br>Oxford, UK	Exclusion criteria included lack of capacity to consent and those who had been a psychiatric inpatient in the last month.<br><br>From feedback, mood and activity monitoring resulted in a change in behavior, which may not reflect accurately in their daily lives. Concerns were also voiced about the context when monitoring mood as reactive changes may be interpreted as relapse. Some participants became overly or underly participatory and aware of the changes in their mood.<br><br>Many participants expressed that the wearable technology may have not accurately captured their activity levels or they were forgetful in wearing it.<br><br>The factors could have a moderate impact on the results and completion rates.
Schwartz et al. (2016) [37]<br><br>Pennsylvania, USA	Bipolar participants could be in any mood state at study entry. While healthy controls did not have a personal or family history of psychiatric illness, it is unclear how they initially entered the research program. It was also unclear how bipolar patients and controls were selected from the research pool and how the number of participants was chosen.<br><br>Discomfort using smartphone technology was an exclusion criterion.<br><br>Impact of above factors difficult to predict as potential confounders from the study population was unclear.
